# A Study of Physical Activity Determinants among High-Risk Hypertensive Filipino and Korean Americans

**DOI:** 10.3390/ijerph16071156

**Published:** 2019-03-31

**Authors:** Aisha Bhimla, Crystal A. Gadegbeku, Yin Tan, Lin Zhu, Ferdinand Aczon, Grace X. Ma

**Affiliations:** 1 Center for Asian Health, Lewis Katz School of Medicine, Temple University, Philadelphia, PA 19140, USA; yin.tan@temple.edu (Y.T.); lin.zhu@temple.edu (L.Z.); grace.ma@temple.edu (G.X.M.); 2 Department of Kinesiology, College of Public Health, Temple University, Philadelphia, PA 19121, USA; 3 Division of Nephrology, Temple University School of Medicine, Philadelphia, PA 19140, USA; crystal.gadegbeku@tuhs.temple.edu; 4 Filipino American Society of South Jersey, Inc., Mount Laurel, NJ 08084, USA; faczon@aol.com; 5 Department of Clinical Sciences, Lewis Katz School of Medicine, Temple University, Philadelphia, PA 19140, USA

**Keywords:** physical activity, Asian American, hypertension

## Abstract

Physical activity (PA) serves a critical role in maintaining health and preventing chronic diseases, though its influence on high-risk Asian American populations is unclear. The purpose of this study was to determine PA levels among Filipino and Korean Americans at high risk of hypertension and to identify sociodemographic and health-related factors associated with PA levels in these populations. A cross-sectional survey was administered to 137 participants in the Greater Philadelphia Area. Data was collected on PA levels, sociodemographic factors, and health factors. Multinomial logistic regression was conducted to determine predictors associated with low, moderate, and high PA and predictive probabilities were calculated for interaction terms, incorporating ethnicity and blood pressure variables. Overall, 42.33% of participants belonged to the moderately active PA group and 21.90% belonged to the highly active group. In the final multinomial regression model, it was found that having gone to college increased the odds of being in the moderately active PA group (coef. = 1.96, *p* = 0.034), while having high blood pressure reduced the odds of being in the moderately active PA group (coef. = −2.21, *p* = 0.022). Lastly, being Korean versus Filipino reduced the odds of being in the highly active category (coef. = −2.89, *p* = 0.035). Based on predictive probabilities, Koreans and Filipinos with high blood pressure were more likely to belong in the low active PA category (52.31% and 46.33%). These findings highlight the need for culturally relevant PA interventions for promoting and increasing PA levels to prevent and manage hypertension among these populations.

## 1. Introduction

Filipinos and Korean Americans constitute two important Asian immigrant populations in the United States (U.S.). Within the last decade, due to increased awareness of disparities when disaggregating Asian Americans into subgroups, their health status has become a public health priority in the U.S. [1]. Highlighted disparities in these groups include a high prevalence of chronic disease. Filipinos face high rates of heart disease, type 2 diabetes, and hypertension compared to non-Hispanic Whites and other Asian American subgroups [2,3,4,5,6]. Additionally, Koreans face higher rates of hypertension, hypocholesteremia, cancer, and diabetes [7,8]. Hypertension, or high blood pressure, is a common chronic disease reported in Filipinos and Korean Americans and frequently is associated with poor dietary and lifestyle factors, including physical inactivity and elevated stress [1,9,10,11,12,13]. Studies investigating hypertension prevalence in these groups found rates ranging from 48−67.5% in Filipinos and 30.8−53% in Koreans [8,14,15,16,17,18]. Hypertension prevention and management are important issues that require greater attention in these populations.

It is well established that physical activity (PA) is a critical component of a healthy lifestyle and plays an important role in the prevention of chronic conditions, including cardiovascular disease, hypertension, specific cancers, hyperlipidemia, and type 2 diabetes [19]. However, few studies have examined PA levels in Asian Americans, and thus little is known regarding PA behaviors in these populations and what factors contribute to regular participation in PA [20]. Moreover, few studies have explored the impacts of sociodemographic and health factors to identify disparities in PA participation specifically in Koreans and Filipinos, despite the potential of such studies to help fuel progress toward the development of culturally relevant interventions. Of recent studies, data suggest that Asian Americans have a reduced likelihood of meeting adequate PA levels [21,22]. A cross-sectional study conducted in two U.S. metropolitan areas, New York City (NYC) and Los Angeles County, found that Asian Americans were half as likely than other racial and ethnic groups to meet the weekly PA guidelines [22]. Of those studies that investigated physical activity specifically in Filipino Americans, only one quarter to one third of Filipinos had met the recommended exercise guidelines of 150 minutes of moderate PA or vigorous PA per week [15,16]. Among Koreans, a recent systematic review examining cardiovascular risk factors found rates of physical activity to range from 12.8−13.3% participating in adequate moderate physical activity and 11.6%−58.8% participating in adequate vigorous activity [18]. In a study conducted among Korean Americans living in Philadelphia, only 22% of participants were meeting the Centers for Disease Control (CDC) physical activity guidelines of 150 or more minutes of moderate activity, 75 minutes or more of vigorous activity, or an equivalent combination [8].

Physical activity participation is viewed as an important component of maintaining weight and preventing obesity related chronic disease [23,24]. Substantial evidence has shown that although Asian Americans are less likely to be overweight or obese compared to other ethnic groups, they exhibit higher risk of chronic diseases at lower body mass index (BMI) values due to higher body fat percentage, which overall underestimates obesity risk [5,25,26,27,28]. The World Health Organization Asian BMI proposed modified cutoffs are more applicable to this population [27]. Epidemiological studies among Korean Americans in California found the percentage of individuals who were overweight or obese based on Asian BMI criteria ranged from 46−60.8% [25,28]. Filipinos face the highest rates of obesity among all ethnic groups, with one study finding a prevalence rate of 78.6% [28]. Both Filipinos and Korean Americans have been identified as targeted populations for assessing overweight and obesity status [28]. Acculturation has been shown to be linked to levels of obesity where those who have lived longer in the US exhibit similar risk to those born in the U.S. [29].

The purpose of this study was: (1) to determine PA levels among Filipinos and Korean Americans who are at high risk of hypertension, and (2) to determine what sociodemographic and health-related factors are associated with PA levels among Filipino and Korean Americans. This study presents baseline findings for interventions addressing sodium consumption and PA health education in Koreans and Filipinos in the Greater Philadelphia and Southern New Jersey Areas.

## 2. Materials and Methods 

### 2.1. Study Design and Data Collection

The study data was collected through a cross-sectional survey that was administered to Filipino and Koreans living in the Greater Philadelphia and Southern New Jersey Areas who underwent an educational intervention to reduce sodium consumption and increase PA levels. Measures were taken at baseline and only reported in this paper. The baseline surveys were administered by staff members, with Korean participants receiving a survey in Korean. The survey took 15−20 minutes to complete. Inclusion criteria were: (1) self-identifying as Filipino or Korean, (2) being at least 18 years of age, (3) having resided in the Greater Philadelphia Area in the past year, and (4) not having previously participated in a health educational lifestyle or hypertension intervention.

### 2.2. Participants

Participants were 71 Filipino−Americans and 66 Korean−Americans adults recruited through 5 community-based organizations (CBOs) and churches in the Greater Philadelphia Area from September 2016–May 2017. There was a total number of 137 participants. The mean average blood pressure taken at baseline among our participants for systolic was m = 133.24 mmHg, SD = 17.56 and for diastolic values m = 75.50 mmHg, SD = 9.85. We used the OMRON IntelliSense® Blood Pressure Monitor model HEM 907XL (OMRON HEALTHCARE, Bannockburn, IL, USA) to measure systolic blood pressure and diastolic blood pressure levels. The study coordinator placed the appropriately sized cuff around the bare upper right arm so that the midpoint of the length of the cuff laid over the brachial artery and the mid-height of the cuff was at heart level. Three blood pressure and pulse measurements were taken and the average of the three readings was recorded.

### 2.3. Measures

#### 2.3.1. Physical Activity Levels

Physical activity was measured using the International Physical Activity Questionnaire short form (IPAQ−short). The IPAQ−short is a 7-item self-administered questionnaire used to measure physical activity levels in young and middle-aged adults from various populations [30]. The IPAQ−short determines PA levels undertaken within the past 7 days [30]. For example, “During the last 7 days, on how many days did you walk for at least 10 minutes at a time [30]?” The IPAQ−short has been used in several studies worldwide and is shown to have high reliability (0.66–0.88) [31,32,33].

IPAQ responses were collected and divided by amount of vigorous activity (average minutes per session, average hours per day, number of days per week), amount of moderate activity (average minutes per session, average hours per day, number of days per week), amount of time spent walking (average minutes per session, average hours per day, number of days per week), and sitting time. Based on the amount of vigorous, moderate, and walking activity levels, calculations were expressed in metabolic equivalents (MET). Based on the MET-scoring, vigorous PA is equal to vigorous weekly PA expenditure × 8 METs, moderate PA is equal to moderate weekly PA expenditure × 4 METs, and walking PA is equal to walking weekly PA expenditure × 3.3 METs (Table 1). Participants were then categorized as being low, moderate, and highly active based on algorithms provided [34]. 

#### 2.3.2. Sociodemographic

The sociodemographic characteristics that were assessed included age, gender, marital status, education level, employment status, and health insurance. Gender was expressed as male or female. Age was categorized as under 40, 40–64, and 65+ years old. Marital status was categorized as “married,” “never married,” or “divorced/separated/widowed.” Education level was categorized into “less than high school” and “some/completed college.” Employment status was categorized as “employed,” “unemployed,” and “not in labor force.” Lastly, participants checked off if they currently have health insurance and whether they have a regular physician to visit.

#### 2.3.3. Health-Related Factors

The presence of high blood pressure was assessed by asking “have you ever been told by your doctor that you have high blood pressure?” Participants were also asked whether they monitored their blood pressure, which is an indicator of engaging in blood pressure control behaviors. 

#### 2.3.4. Body Mass Index (BMI)

BMI was calculated from self-reported height (inches) and weight (lb). The formula was used to determine BMI = weight (lb)/(height (in)^2^) × 703. Asian BMI was used to categorize participants into two categories. A BMI cutoff of less than 22.9 indicated underweight or normal weight, while 23 and above indicated overweight or obese [27]. 

### 2.4. Statistical Analysis

Data from the survey was entered into a statistical analysis program, STATA version 14 (StataCorp LLC, College Station, TX, USA). Descriptive analyses were run for determining the differences in sociodemographic characteristics between physical activity categories. Chi-square tests or Fisher exact tests were ran to examine any differences of these sociodemographic, health, and BMI variables. Multinomial logistic regression was used to determine the sociodemographic and health related predictors of PA, based on three categories. The low active PA category served as the reference group. Four different multinomial regression models were created based on interaction terms between ethnicity and health indicators (high blood pressure, monitoring blood pressure) that were each introduced separately in models 2 and 3 and together with other predictors in model 4. A *p*-value of <0.05 was selected for determining which variables were selected for the multinomial logistic regression and to determine the level of significance of variables. The sample size after list wise deletion was 113 participants. The coefficient (log odds), 95% confidence interval, log likelihood, pseudo r-square, and likelihood ratio (LR) Chi-square were reported. Furthermore, predicted probabilities of PA level among Filipinos and Koreans with and without high blood pressure as well as monitoring and non-monitoring of blood pressure were calculated when other variables were held at the mean value for each group.

## 3. Results

### 3.1. Physical Activity Levels 

Descriptive statistics of mean physical activity levels among the three groups are in Table 2. The mean and standard deviation for metabolic equivalents (METs) were 37.86 (162.51) for the low activity group, 704.43 (1060.41) for the moderate activity group, and 6134.25 (2765.60) for the high activity group.

### 3.2. Descriptive Characteristics among All Participants and by Physical Activity Level Group

Descriptive statistics are presented in Table 3. Around half of the study sample was over 65 years old and above (51.09%) and of Filipino ethnicity (51.82%). The majority of the study sample was female (70.07%), currently married (71.54%), and had some sort of college completed or higher (80.74%). The frequency distributions for ethnicity, gender, marital status, and education were similar between low, moderately, and highly active PA levels.

Regarding health-related factors, slightly more than half of the study sample was in the underweight or normal weight category of Asian BMI (51.13%) and reported having high blood pressure (50.39%). The majority of participants had health insurance (89.39%) and were monitoring their blood pressure (72.39%). The three PA groups differed significantly on the specific health factors. Those who did not have health insurance were more likely (*p* = 0.003) to belong to the low activity category (78.57%) versus those who did have health insurance (32.30%). Additionally, those who did not monitor their blood pressure (*p* = 0.007) were more likely to belong to the low active category (56.76%) versus those who did monitor their blood pressure (27.85%).

### 3.3. Multinomial Logistic Regression and Predictive Probability Analyses

Results of the multinomial logistic regression on PA status are presented in Table 4. In the first model, having health insurance and monitoring of blood pressure were found to increase the odds of being moderately active (coef. = 2.00, *p* = 0.042; coef. = 1.73, *p* = 0.010) and highly active (coef. = 2.43, *p* = 0.045; coef. = 1.85, *p* = 0.019) compared to low active. Compared to the low activity group, having high blood pressure decreased the odds of being moderately active and this was statistically significant (coef. = −1.40, *p* = 0.033). In the second model, having gone to college (coef. = 1.84, *p* = 0.041) and monitoring blood pressure (coef. = 1.98, *p* = 0.006) increased the odds of being in the moderately active PA group, whereas having high blood pressure decreased the odds of being in the moderately active PA group (coef. = −2.61, *p* = 0.005). When introducing the interaction term into the second model, ethnicity and having/not having high blood pressure (Ethnic*High BP), Koreans who reported having high blood pressure had a higher odds of being in the moderately active PA group (coef. = 2.25, *p* = 0.046) versus Filipinos with high blood pressure. Among those in the highly active PA group, being Korean versus Filipino had a lower odds associated with belonging to this category (coef. = −2.32, *p* = 0.021). With respect to health indicators, having high blood pressure decreased the odds (coef. = −2.00, *p* = 0.044) and monitoring blood pressure increased the odds (coef. = 2.12, *p* = 0.012) of belonging to the highly active PA group. In the third model, introducing the interaction terms ethnicity and monitoring blood pressure (Ethnic*Monitor BP) found that having high blood pressure remained a significant predictor in being moderately active (coef. = −1.40, *p* = 0.036). In the fourth model, having gone to college increased the odds of being in the moderately active PA group (coef. = 1.96, *p* = 0.034), while having high blood pressure reduced the odds of being in the moderately active PA group (coef. = −2.21, *p* = 0.022). Lastly, being Korean versus Filipino had reduced the odds of being in the high active category (coef. = −2.89, *p* = 0.035). Both interaction terms did not remain significant in the final model. In the final model, the log likelihood was −102.96, pseudo r-square was 0.1830 and the likelihood ratio was 46.13 (*p* = 0.0088).

**Figure 1:**
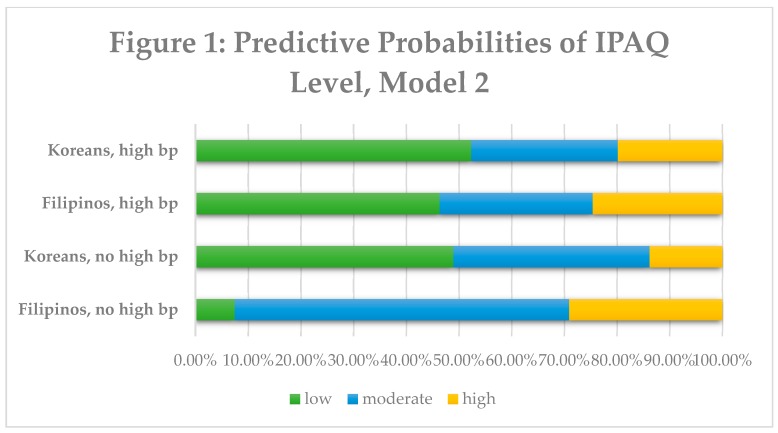
There are four predictive probabilities presented based on ethnicity and high blood pressure status. Physical activity level (%) is represented in the graph; green represents low activity group, blue represents moderate activity group and yellow represents the high activity group.

**Figure 2:**
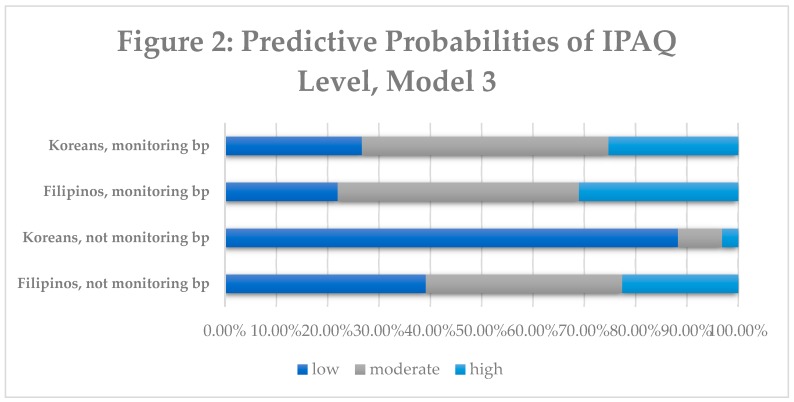
There are four predictive probabilities presented based on ethnicity and monitoring of blood pressure. Physical activity level (%) is represented in the graph; dark blue represents low activity group, grey represents moderate activity group and light blue represents the high activity group.

Predictive probabilities of PA level are displayed in Figure 1 and Figure 2 below. In the second model, Koreans with high blood pressure were more likely to belong in the low active PA category (52.31%), followed by moderate and high. Filipinos with high blood pressure were more likely to belong into the low active PA category (46.33%). Koreans without high blood pressure were more likely to belong to the low active category (48.97%), whereas Filipinos without high blood pressure were more likely to belong to the moderate PA category (63.47%). In the third model, Koreans who monitored their blood pressure were more likely to belong to the moderately active category (48.03%) as well as Filipinos monitoring their blood pressure (46.98%). However, when comparing Koreans not monitoring their blood pressure versus Filipinos not monitoring their blood pressure, a significant amount of Koreans were more likely to belong to the low active category (88.30% vs. 39.14%).

## 4. Discussion

This study demonstrates the differences in sociodemographic and health related factors that influence PA levels among Koreans and Filipino Americans. We also investigated whether there were any interactive effects of ethnicity and health behaviors on physical activity level. In our final regression model, education level, ethnicity, and high blood pressure status were significant predictors of being moderately or highly active. Furthermore, predicted probabilities illustrated that those who had high blood pressure were more likely to belong to the low physical activity group. 

Overall, the percentage of participants in our study who reported engaging in moderately active (42.33%) and highly active (21.90%) PA levels was higher than the general U.S. population but similar to studies conducted among other Asian groups in the US [21,22]. In our sample, 64.23% of Filipinos and Koreans combined were moderately/highly active in the US, whereas generally only 50% of Americans met the moderate aerobic PA guidelines [35]. In another study measuring physical activity levels among Filipino and Koreans in California, 73.8% from each ethnic group were meeting the American College of Sport Medicine (ACSM) physical activity levels of 450 metabolic equivalents (METs) or more per week [20]. Self-reported PA levels should be interpreted with caution, as other studies conducted on Filipinos and Koreans measuring PA levels found that individuals were overreporting when asked to fill out their activity in a survey, versus when more objective measures were utilized [36,37,38]. However, our study used a validated PA tool that has been utilized in several other countries and in diverse settings [31]. 

With regards to the multinomial regression analysis and ethnic differences in physical activity levels, Filipino Americans were more likely to be moderately to highly active compared to Koreans. In the few studies that disaggregated Asian Americans by groups, Korean Americans were generally less physically active compared to Filipinos [18,39]. A representative study of Asian Americans in California examining health risk behaviors among various Asian American groups found that among Koreans, 45% of men and 48% of women participated in moderate PA and 28% of men and 16% of women participated in vigorous PA, which was lower than Filipinos, where 52% of men and 56% of women participated in moderate PA and 30% of men and 26% of women participated in vigorous PA [39]. Differences may lie in cultural, environmental, and psychosocial factors that influence participation levels among these two groups. Level of acculturation, or the cultural and psychological changes associated with exposure to a different culture, varies among Koreans versus Filipinos and can affect changes in lifestyle behaviors due to years of residence in the U.S. [40]. A study carried out in California indicated that higher levels of acculturation among Korean Americans was associated with engaging in more PA [41]. Filipino Americans have lived in the U.S. for long periods and have unique lifestyle behaviors, which reflect both American and Filipino culture [42]. Understanding how culture influences PA can provide a background to developing culturally tailored interventions for these high-risk groups. 

Additionally, participants with a higher education level indicated by having attended college were more likely to belong to the moderately active PA category. This is consistent with the literature where higher socioeconomic status, specifically with education being a proxy measure, is associated with higher levels of PA [43]. Among Asian Americans who studied in NYC, Los Angeles, and Houston, those with lower education levels were less likely to meet the CDC PA guidelines [21,22]. In a large study on Asian American subgroups in California, social determinants of PA, including living above the poverty level but not education levels, were associated with higher levels of PA in Asian Americans [20]. We did not examine any differences that were age or gender related in PA levels among our sample. However, a study on the California Health Interview Survey found that middle-aged Filipino and Korean females were significantly less likely to meet the ACSM guidelines [22]. It is important to consider socioeconomic status when conducting PA interventions among these specific ethnic groups as there is an evident disparity in PA levels among those with lower education levels and among Korean versus Filipino Americans. 

With respect to health conditions, having high blood pressure decreased the odds of having moderate PA levels. Although our study was one of the first to investigate relationships between health factors and PA level among Filipino and Korean Americans, some studies have examined self-rated health, finding that Asian Americans with poor/fair self-rated health had a lower number of reported PA days and were half as likely to meet sufficient PA levels [21]. PA has the potential to prevent and manage hypertension, illustrating the need to address PA interventions among those who have hypertension in order to further control their disease. Studies that have examined blood pressure control behaviors found that Filipinos and Koreans with hypertension or poor health management (e.g., lack of blood pressure screening) exhibited lower PA levels and less likeliness to meet PA guidelines, as well as only 20% of Korean women had awareness of PA as a lifestyle behavior for controlling hypertension [44,45,46]. In our study, Koreans and Filipinos who reported monitoring their blood pressure were more likely to belong to the moderately active PA category (48.03% and 46.98%, respectively). Thus, those who have their blood pressure under control may engage in healthier lifestyle behaviors, which includes regular participation in PA. 

### Limitations

Our study faced limitations. First, our study was cross-sectional in nature, preventing causal conclusions to be drawn between the selected factors and their impact on PA levels among Filipino and Korean participants who are at risk of hypertension. Secondly, PA, hypertension status, and blood pressure monitoring was based on self-reports, which in the past have not been an accurate measure and can lead to over- or under-reporting. Future studies can incorporate more objective measures for PA in this population, such as accelerometers or pedometers that have reported high validity and reliability. Additionally, clinical measures administered by a health care professional can be taken to measure hypertension status and BMI. Furthermore, the study did not look at types of PA and leisure activities that these populations participate in, which are important indicators of PA perception. Lastly, future studies can assess the psychological and environmental factors which impact participation among Asian Americans, as these are important factors identified in the literature that were not collected in our study. For example, when examining specific barriers to Filipino and Korean Americans, previous studies have found that social isolation from one’s own ethnicity, presence of a chronic illness, low environmental resources/access, and low self-efficiency reduce the ability to participate in PA [20,36,47,48,49,50]. These psychosocial factors are important to developing future interventions to increase PA levels in ethnic minority populations. 

## 5. Conclusions

This study demonstrated that for Filipino Americans, higher education levels and an absence of high blood pressure were predictors of meeting moderate/high physical activity levels. 

Future studies can be conducted to test the effectiveness of culturally relevant PA interventions that take sociodemographic factors into consideration. Targeted health education and cultural adaptation of PA can aid in increasing PA levels among these groups, facilitating the prevention and management of hypertension and other chronic diseases.

## Figures and Tables

**Table 1:** International physical activity questionnaire (IPAQ) scoring guidelines and physical activity (PA) classification criteria.

**PA Scoring**
Vigorous PA = 8 × (vigorous activity per day) × (minutes of vigorous activity per day)
Moderate PA = 4 × (moderate activity per day) × (minutes of moderate activity per day)
Walking PA = 3.3 × (walking per day) × (minutes of walking per day)
Total PA score = Vigorous PA + Moderate PA + Walking PA
**PA Classification Criteria**
*Category 1: Low active*
No activity is reported or some activity is reported but not enough to meet the other two categories.
*Category 2: Moderately active*—any of the following criteria:
3 or more days of vigorous activity of at least 20 minutes per day
5 or more days of moderate intensity activity or walking of at least 30 minutes per day
5 or more days of any combination of walking, moderate-intensity, and vigorous activities achieving a minimum of at least 600 MET−min/week.
*Category 3: Highly active*—any of the following:
Vigorous activity on at least 3 days and accumulating at least 1500 MET−minutes/week, 7 or more days of any combination of walking, moderate, or vigorous activity achieving a minimum of at least 3000 MET−minutes/week.

**Table 2:** Physical activity levels among participants (n = 137).

Physical Activity Levels (METs)	Low Category	Moderate Category	High Category	Total
Mean, standard deviation	37.86 ± 162.51	703.43 ± 1060.41	6134.25 ± 2765.60	1654.61 ± 2804.59

**Table 3:** Demographic and acculturative characteristics among all participants and by physical activity level group (n = 137).

Variable	Low	Moderate	High	All
	n (%)	n (%)	n (%)	n (%)
**Age (years)**				
Under 40	3 (37.5)	4 (50)	1 (12.5)	8 (5.84)
40–64	26 (44.07)	17 (28.81)	16 (27.12)	59 (43.07)
65+	20 (28.57)	37 (52.86)	13 (18.57)	70 (51.09)
				*p* = 0.090 ^‡^
**Ethnicity**				
Filipino	20 (28.17)	33 (46.48)	18 (25.35)	71 (51.82)
Korean	29 (43.94)	25 (37.88)	12 (18.18)	66 (48.18)
				*p* = 0.151
**Gender**				
Male	12 (29.27)	18 (43.90)	11 (26.83)	41 (29.93)
Female	37 (38.54)	40 (41.67)	19 (19.79)	96 (70.07)
				*p* = 0.503
**Marital status**				
Married	34 (36.56)	36 (38.71)	23 (24.73)	93 (71.54)
Never married	4 (50.00)	3 (37.50)	1 (12.50)	8 (6.15)
Divorced/widowed/separated	10 (34.48)	15 (15.72)	4 (13.79)	29 (22.31)
				*p* = 0.574 ^‡^
**Education**				
≤High school	12 (46.15)	10 (38.46)	4 (15.38)	26 (19.26)
≥College	37 (33.94)	46 (42.20)	26 (23.85)	109 (80.74)
				*p* = 0.447
**Asian BMI**				
Underweight or normal weight	22 (32.35)	30 (44.12)	16 (25.53)	68 (51.13)
Overweight or obese	25 (38.46)	26 (40.00)	14 (21.54)	65 (48.87)
				*p* = 0.762
**Have health insurance**				
Yes	38 (32.30)	51 (43.22)	29 (24.58)	118 (89.39)
No	11 (78.57)	2 (14.29)	1 (7.14)	14 (10.61)
				*p* = 0.003 * ^‡^
**Have high blood pressure**				
Yes	23 (35.94)	26 (40.63)	15 (23.44)	65 (50.39)
No	24 (39.92)	26 (40.00)	15 (23.08)	64 (49.61)
				*p* = 0.993
**Monitor blood pressure**				
Yes	27 (27.84)	45 (46.39)	25 (25.77)	97 (72.39)
No	21 (56.76)	11 (29.73)	5 (13.51)	37 (27.61)
				*p* = 0.007 *

* $\chi^{2}$(df) *p*-value < 0.05, ^‡^ Fisher exact test statistics.

**Table 4:** Multinomial logistic regression analysis of demographic and acculturative factors and PA among Filipino and Korean Groups (n = 113).

Variable	Model 1		Model 2		Model 3		Model 4	
	Moderate Act.	High Act.	Moderate Act.	High Act.	Moderate Act.	High Act.	Moderate Act.	High Act.
	(vs. Low)	(vs. Low)	(vs. Low)	(vs. Low)	(vs. Low)	(vs. Low)	(vs. Low)	(vs. Low)
	Coefficient	Coefficient	Coefficient	Coefficient	Coefficient	Coefficient	Coefficient	Coefficient
	(95% CI)	(95% CI)	(95% CI)	(95% CI)	(95% CI)	(95% CI)	(95% CI)	(95% CI)
**Age**								
Under 40	Ref.	Ref.	Ref.	Ref.	Ref.	Ref.	Ref.	Ref.
40–64	−1.16 (−3.31–0.98)	−0.18 (−3.04–2.68)	−1.38 (−3.54–0.78)	−0.42 (−3.33–2.48)	−1.13 (−3.31–1.05)	−0.16 (−3.12–2.80)	−1.29 (−3.48–0.89)	−0.34 (3.30–2.63)
65+	−0.64 (−2.95–1.66)	−1.14 (−4.16–1.87)	−0.74 (−3.05–1.57)	−1.23 (−4.26–1.81)	−0.50 (−2.87–1.86)	−0.99 (−4.12–2.13)	−0.62 (−2.97–1.73)	−1.10 (−4.21–2.00)
**Ethnic**								
Filipino	Ref.	Ref.	Ref.	Ref.	Ref.	Ref.	Ref.	Ref.
Korean	−0.43 (−1.71–0.85)	−1.17 (−2.63–0.28)	−1.55 (−3.28–0.19)	−2.32 (−4.30–−0.35) *	−1.66 (−3.66–0.33)	−2.62 (−5.26–0.01)	−1.95 (−4.01–0.12)	−2.89 (−5.57–−0.21) *
**Gender**								
Male	Ref.	Ref.	Ref.	Ref.	Ref.	Ref.	Ref.	Ref.
Female	−0.46 (−1.69–0.76)	−0.88 (−2.24–0.47)	−0.34 (−1.58–0.91)	−0.78 (−2.16–0.59)	−0.15 (−1.43–1.12)	−0.58 (−1.97–0.80)	−0.20 (−1.48–1.09)	−0.63 (−2.04–0.77)
**Marital status**								
Married	Ref.	Ref.	Ref.	Ref.	Ref.	Ref.	Ref.	Ref.
Never married	−0.96 (−3.12–1.19)	−2.09 (−4.85–0.68)	−1.21 (−3.47–1.05)	−2.25 (−5.12–0.62)	−0.92 (−3.12–1.27)	−1.98 (−4.81–0.85)	−1.10 (−3.37–1.16)	−2.13 (−5.02–0.76)
Divorced/Widowed/Separated	0.75 (−0.62–3.13)	0.15 (−1.50–1.80)	0.87 (−0.54–2.28)	0.29 (−1.40–1.97)	0.51 (−0.90–1.93)	−0.08 (−1.77–1.62)	0.69 (−0.77–2.15)	0.10 (−1.63–1.84)
**Education**								
≤High school	Ref.	Ref.	Ref.	Ref.	Ref.	Ref.	Ref.	Ref.
≥College	1.33 (−0.30–2.96)	0.41 (−1.42–2.24)	1.84 (0.08–3.60) *	0.95 (−1.02–2.92)	1.79 (−0.002–3.58)	0.96 (−1.03–2.95)	1.96 (0.15–3.78) *	1.13 (−0.91–3.16)
**Health insurance**								
No	Ref.	Ref.	Ref.	Ref.	Ref.	Ref.	Ref.	Ref.
Yes	2.00 (0.08–3.92 )*	2.43 (0.05–4.81) *	1.67 (−0.35–3.70)	2.09 (−0.45–4.63)	1.98 (−0.07–4.04)	2.44 (−0.03–4.92)	1.73 (−0.34–3.79)	2.17 (−0.39–4.72)
**Asian BMI**								
Underweight/normal	Ref.	Ref.	Ref.	Ref.	Ref.	Ref.	Ref.	Ref.
Overweight/obese	−0.66 (−1.71–0.40)	−0.41 (−1.58–0.75)	−0.77 (−1.86–0.32)	−0.55 (−1.76–0.65)	−0.70 (−1.78–0.39)	−0.48 (−1.67–0.72)	−0.75 (−1.85–0.34)	−0.54 (−1.75–0.67)
**High blood pressure (BP)**								
No	Ref.	Ref.	Ref.	Ref.	Ref.	Ref.	Ref.	Ref.
Yes	−1.40 (−2.69–−0.11) *	−0.78 (−2.18–0.62)	−2.61 (−4.42–−0.79) *	−2.00 (−3.94–−0.05) *	−1.40 (−2.72– −0.09) *	−0.80 (−2.21–0.61)	−2.21 (−4.10– −0.32) *	−1.58 (−3.58–0.41)
**Monitor blood pressure (BP)**								
No	Ref.	Ref.	Ref.	Ref.	Ref.	Ref.	Ref.	Ref.
Yes	1.73 (0.41–3.05) *	1.85 (0.31–3.38) *	1.98 (0.04–4.46) *	2.12 (0.47–3.76) *	0.78 (−0.89–2.46)	0.89 (−0.96–2.74)	1.29 (−0.65–3.23)	1.39 (−1.18–4.39)
Ethnic*HighBP (Kor, yes)			2.25 (0.57–3.40) *	2.29 (−0.18–4.76)			1.62 (−0.88–4.13)	1.61 (−0.73–3.51)
Ethnic*MonitorBP (Kor, yes)					2.14 (−0.25–4.52)	2.39 (−0.67–5.46)	1.25 (−1.48–3.99)	1.49 (−1.94–4.93)
**Constant**	−1.66 (−5.32–2.00)	−1.70 (−6.24–2.84)	−1.15 (−4.97–2.68)	−1.19 (−5.94–3.56)	−1.71 (−5.65–2.23)	−1.84 (−6.59–2.92)	−1.30 (−5.26–2.66)	−1.39 (−6.23–3.46)
**Log likelihood**	−105.99225		−103.4861		−103.89338		−102.95915	
**Pseudo r-square**	0.1589		0.1788		0.1756		0.183	
**LR Chi−square (df)**	40.06 (22), *p* = −0.0106		45.07 (24), *p* = 0.0057		44.26 (24), *p* = 0.0071		46.13 (26), *p* = 0.0088	

* *p*-value < 0.05.
